# Supplementary material for: DNA Methylation Patterns Correlate with the Expression of SCNN1A, SCNN1B, and SCNN1G (Epithelial Sodium Channel, ENaC) Genes
Source: Int J Mol Sci. 2021 Apr 4;22(7):3754. doi: 10.3390/ijms22073754 (PMC8038451; doi:10.3390/ijms22073754)
Supplement: Supplementary file 1 [file ijms-22-03754-s001.pdf]

Table S1 - Primers used for qualitative endpoint PCR.

Gene	Forward primer	Reverse primer	Amplicon length	T_a
GAPDH	5'-CCCTTCATTGACCTCAACTACATGA-3'	5'-TGGGATTTCCATTGATGACAAGC-3'	116bp	62°C
SCNN1A	5'-GCTGATAACCAGGACAAAACACAA-3'	5'-CGTCGCTGGGCAGGAA-3'	68bp	60°C
SCNN1B	5'-GAGCCCTGCAACTACCGGA-3'	5'-GCCGAAGGAAGTGCCTTCTC-3'	101bp	60°C
SCNN1G	5'-GCCCTGAAGTCCCTGTATGG-3'	5'-CGGTGGGAGAATCTAGGCTG-3'	101bp	60°C
CFTR (Ex6-Ex7)	5'-TGGGAGTTGTTACAGGCGTCTGCC-3'	5'-AGGGAAATTGCCGAGTGACCGC-3'	421bp	60°C
CFTR (Ex8-Ex10)	5'-ACAAAAGCAAGAATATAAGACATTG-3'	5'-GAATGAAATTCTTCCACTGTGC-3'	346bp	60°C
CFTR (Ex11-Ex13)	5'-ACACTGAGTGGAGGTCAACG-3'	5'-CCATTTTAGAAGTGACCAAAATCC-3'	184bp	60°C
CFTR (Ex11-Ex15)	5'-ACACTGAGTGGAGGTCAACG-3'	5'-AGCAAAGTGTCGGCTACTCC-3'	1142bp	60°C

Table S2 - Primers used for quantitative real time PCR.

Gene	Forward primer	Reverse primer	Amplicon length	T_a
Actin-β	5'-GCCGGGACCTGACTGACTA-3'	5'- TGGTGATGACCTGGCCGT -3'	204bp	60°C
SCNN1A	5'-GCTGATAACCAGGACAAAACACAA-3'	5'-CGTCGCTGGGCAGGAA-3'	68bp	60°C
SCNN1B	5'-GAGCCCTGCAACTACCGGA-3'	5'-GCCGAAGGAAGTGCCTTCTC-3'	101bp	60°C
SCNN1G	5'-GCCCTGAAGTCCCTGTATGG-3'	5'-CGGTGGGAGAATCTAGGCTG-3'	101bp	60°C
CFTR (Ex10-Ex11)	5'-AAGCGTCATCAAAGCATGCC-3'	5'TTGCTCGTTGACCTCCACTCA-3'	110bp	60°C

Table S3 - Primers used for HpaII / PCR.

A) *SCNN1A* gene.

Region	Forward primer	Reverse primer	Amplicon length	T_a
a	5’-CAAGATTCAGCAGAGATGACACC-3’	5’-TCCTGGTCCCTCCTCTTTCC-3’	875bp	66-59°C
b	5’-CTAGCTCCTGGAAGCACACTTG-3’	5’-TGTGTCCTGATTCTGTCTCTGC-3’	711bp	66-59°C
c	5’-AGAGGAGAGGCCGTTGTTGTAGG-3’	5’-GCTGAAGTACTCTCCGAAAAGC-3’	636bp	66-59°C
control	5’-ATCAACCTCAACTCGGACAAGC-3’	5’-GTGCTAGGATGGATTCACTGG-3’	265bp	66-59°C

B) *SCNN1B* gene.

Region	Forward primer	Reverse primer	Amplicon length	T_a
a	5’-TGAGTCCAGGAGTTCCAGACC-3’	5’-CCACGAATATGTCCACAGACC-3’	924bp	66-59°C
b	5’-CAGCTCCCCAAAGGTAAACACC-3’	5’-ATTCATGGGTCCGTATGTGAGC-3’	655bp	66-59°C
c	5’-ATTTGAACCCAGGCAGTCC-3’	5’-ACACAGCTCAATGGGTAGGC-3’	487bp	66-59°C
d	5’-CCAGCCTACATGGTGAAACC-3’	5’-CCCATCGGTAGGCATTATCC-3’	355bp	66-59°C
control	5’-AGTTCAGGCAATTCCCTTCC-3’	5’-GGCCATCTCCAGGTCTCC-3’	188bp	66-59°C

C) *SCNN1G* gene.

Region	Forward primer	Reverse primer	Amplicon length	T_a
a	5’-AGACGCGTGGATCACCTG-3’	5’-AAGGGTCCAAGGCTCGTG-3’	871bp	66-59°C
b	5’-TGGAACCGAAAGGTGAGTTC-3’	5’-AGATTTGCCCCAAGTCTAGC-3’	670bp	66-59°C
control	5’-GTGAAAATTAAATGAGGTGACAGC-3’	5’-ACCTCCTCCCTCACTACAATCC-3’	485bp	66-59°C

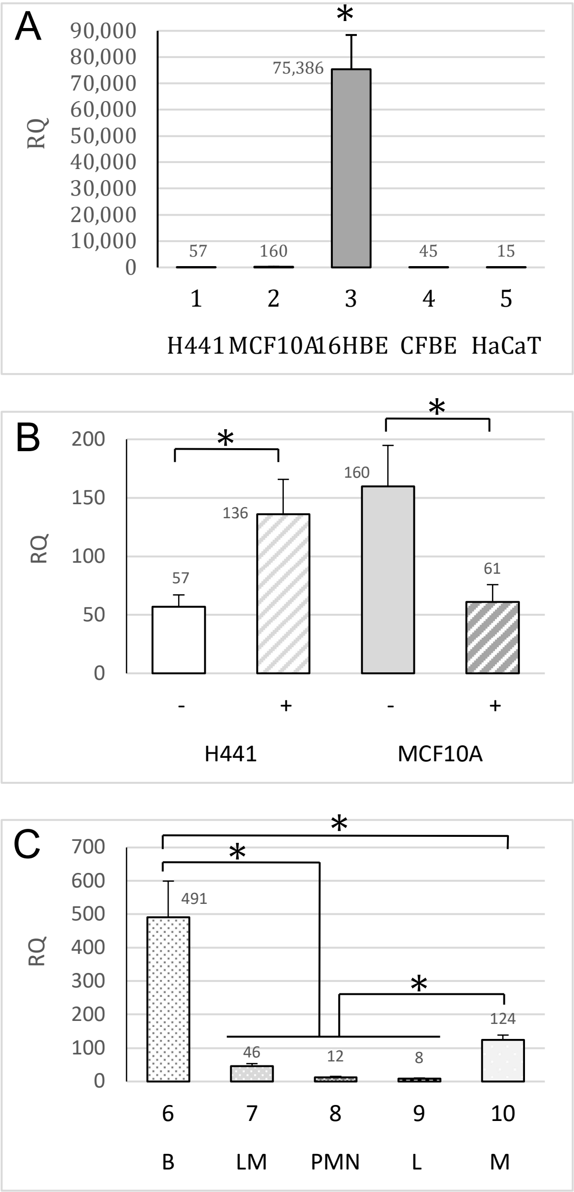

Figure S1 – Quantitative *CFTR* gene expression analysis by real time PCR.
A) Results represent the expression of *CFTR* in H441, MCF10A, 16HBE, CFBE, HaCaT cell lines (respectively from 1 to 5). **B)** Results with (+) and without (-) dexamethasone treatment are shown for H441 and MCF10A cell lines. **C)** Results for nasal brushing (B), lymphocytes/monocytes (LM), granulocytes (PMN), lymphocytes (L) and monocytes (M) are shown (from 6 to 10). A relative quantification (RQ) is reported on y-axis, as fold changes in respect to *SCNN1B* expression in CFBE (Figure 1, panel D, column 4 of main text) used as reference condition (the numbers above the bars are the exact RQ values). For panels A and C, ANOVA $p<0.01$; for panel A the single * indicates the only statistically significant difference following Bonferroni's multiple comparison test (*$p<0.01$); for panel C the statistically significant differences between specific conditions following Bonferroni's multiple comparison test are as indicated (*$p<0.01$). For panel B, Student's t-test of all dexamethasone treated cells (+) as compared to respective untreated cells (-) *$p<0.01$.

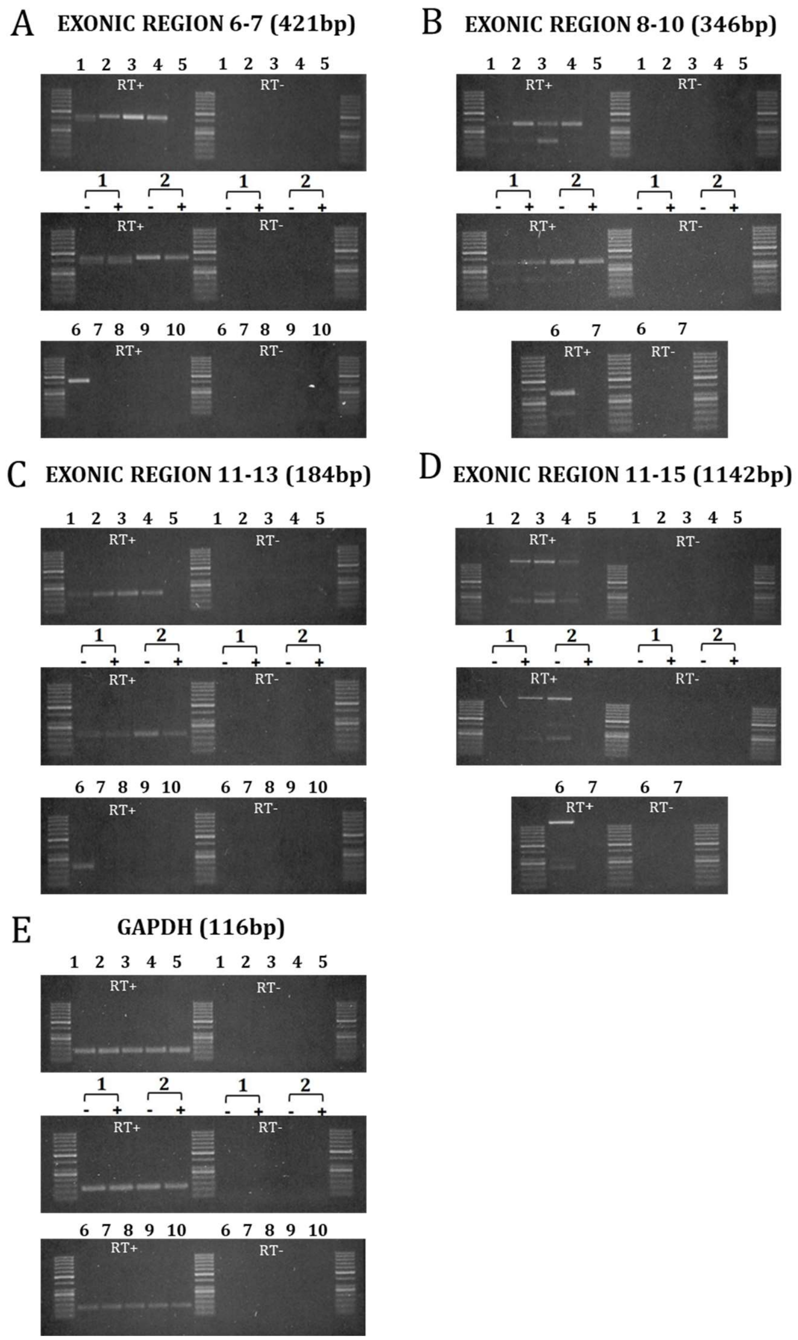

Figure S2 – Qualitative *CFTR* gene expression analysis by endpoint PCR.
Results represent the expression of *CFTR* in indicated cell lines (1-5) and *ex vivo* samples (6-10). The analysis was performed by a qualitative endpoint PCR protocol by studying the 4 CFTR exonic regions indicated (with the size of amplicons showed in base pairs (bp)). For some cell lines (1, 2) results with (+) and without (-) dexamethasone treatment are shown. Panels from A to D refer to *CFTR* expression analysis after 38 cycles of PCR amplification protocol. Panel E refers to *GAPDH* expression analysis after 28 cycles of PCR amplification protocol. In every panel: 1 = H441, 2 = MCF10A, 3 = 16HBE, 4 = CFBE, 5 = HaCaT, 6 = nasal brushing, 7 = lymphocytes + monocytes, 8 = granulocytes, 9 = lymphocytes, 10 = monocytes. The first and last lane of each panel contain the DNA ladder described in Materials and Methods.

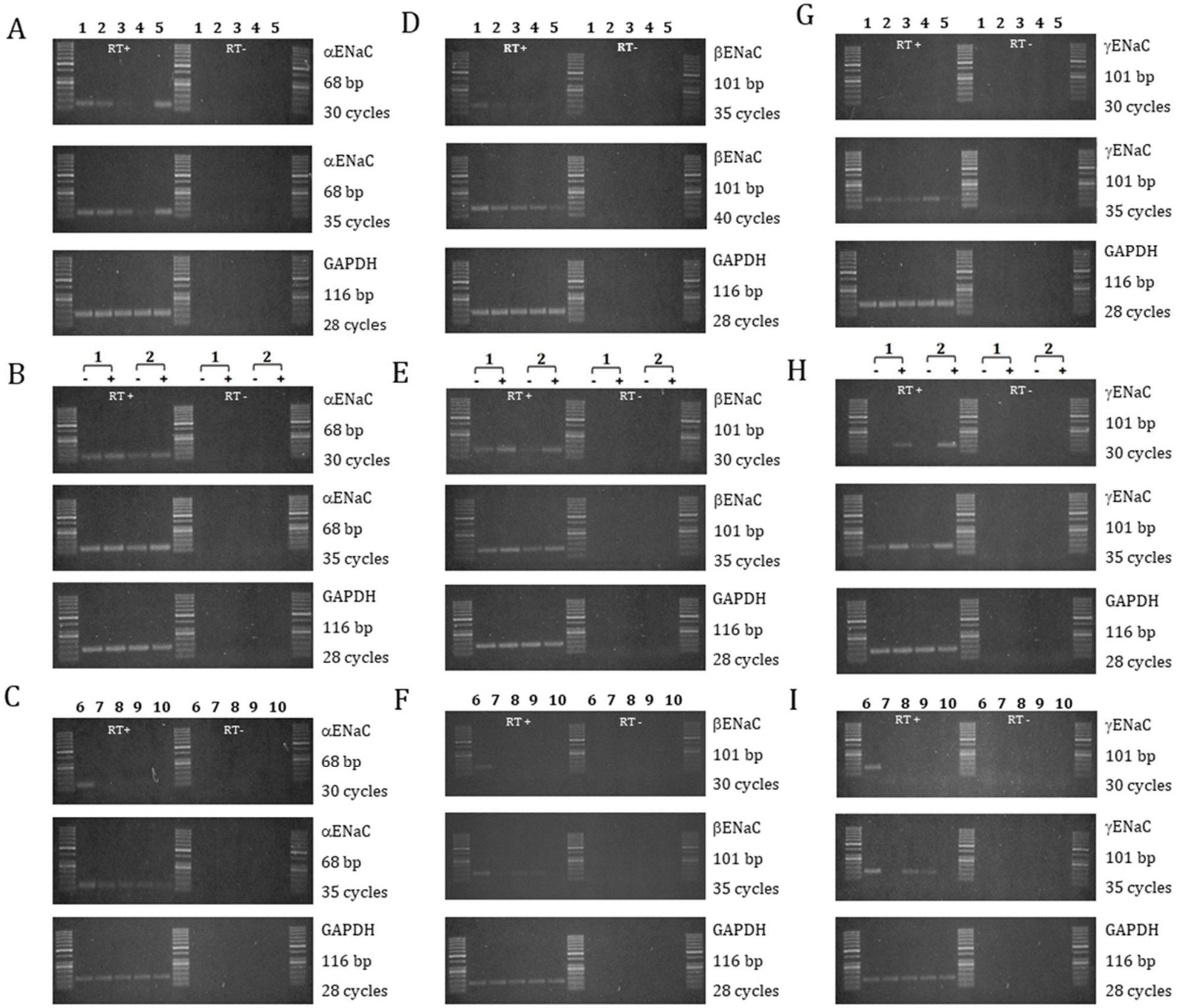

Figure S3 – Qualitative expression analysis of ENaC genes by endpoint PCR.
Panels refer to the indicated number of cycles of PCR amplification protocol for *SCNN1A* (panels A, B and C*), SCNN1B* (panels D, E and F) and *SCNN1G* (panels G, H and I) genes. *GAPDH* gene was analyzed after 28 cycles of PCR amplification. The size of amplicons in base pairs (bp) is shown on the right of each panel. Cell lines (1-5, panels A, D and G), H441 and MCF10A treated (+) or untreated (-) with dexamethasone (panels B, E and H) and *ex vivo* samples (6-10, panels C, F and I) are indicated as follows. In every panel: 1 = H441, 2 = MCF10A, 3 = 16HBE, 4 = CFBE, 5 = HaCaT, 6 = nasal brushing, 7 = lymphocytes + monocytes, 8 = granulocytes, 9 = lymphocytes, 10 = monocytes. The first and last lane of each panel contain the DNA ladder described in Materials and Methods.

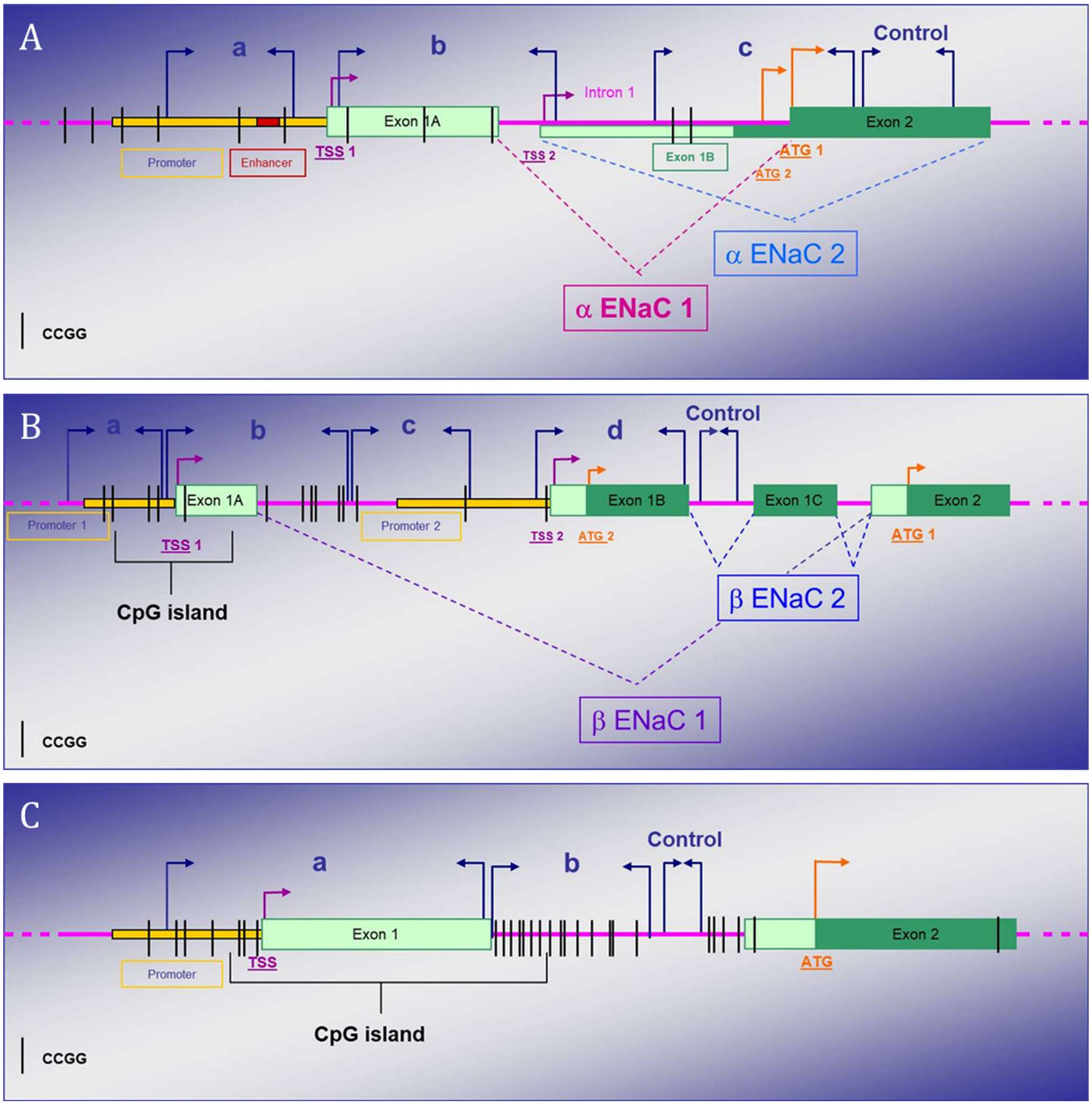

Figure S4 – Schematic representation of 5'-flanking regions of ENaC genes.
A) *SCNN1A* gene. Blue arrows indicate the 4 analyzed regions (a, b, c, Control). Black lines indicate CCGG sites. The entire region analyzed consists of 2926 base pairs of the 5'-flanking region of *SCNN1A* gene, covering a total of 7 CCGG sites. **B) *SCNN1B* gene.** Blue arrows indicate the 5 analyzed regions (a, b, c, d, Control). Black lines indicate CCGG sites. The entire region analyzed consists of 3842 base pairs of the 5'-flanking region of *SCNN1B* gene, covering a total of 14 CCGG sites. The position of the CpG island is shown. **C) *SCNN1G* gene.** Blue arrows indicate the 3 analyzed regions (a, b, Control). Black lines indicate CCGG sites. The entire region analyzed consists of 2537 base pairs of the 5'-flanking region of *SCNN1G* gene, covering a total of 21 CCGG sites. The position of the CpG island is shown.
